# Tumor-Derived LIF Promotes GDF15-Driven Cachexia and Adverse Outcomes in Gastric Cancer

**DOI:** 10.3390/cells15040355

**Published:** 2026-02-16

**Authors:** Cristina Di Giorgio, Nicola Natalizi, Maria Rosaria Sette, Martina Bordoni, Benedetta Sensini, Ginevra Lachi, Eleonora Giannelli, Francesca Paniconi, Luigi Cari, Silvia Marchianò, Michele Biagioli, Elva Morretta, Maria Chiara Monti, Bruno Charlier, Fabrizio Dal Piaz, Angela Zampella, Eleonora Distrutti, Luigina Graziosi, Annibale Donini, Stefano Fiorucci

**Affiliations:** 1 Department of Medicine and Surgery, University of Perugia, 06132 Perugia, Italy; nicola.natalizi@dottorandi.unipg.it (N.N.); mariarosaria.sette@libero.it (M.R.S.); martina.bordoni@unipg.it (M.B.); benedetta.sensini@dottorandi.unipg.it (B.S.); ginevra.lachi@dottorandi.unipg.it (G.L.); eleonora.giannelli@unipg.it (E.G.); francesca.paniconi@dottorandi.unipg.it (F.P.); luigi.cari@unipg.it (L.C.); silvia.marchiano@unipg.it (S.M.); michele.biagioli@unipg.it (M.B.); luigina.graziosi@unipg.it (L.G.); annibale.donini@unipg.it (A.D.); 2 Department of Pharmacy, University of Naples Federico II, 80131 Naples, Italy; elva.morretta@unina.it (E.M.); mariachiara.monti@unina.it (M.C.M.); azampell@unina.it (A.Z.); 3 Farmacologia Clinica, A.O.U. San Giovanni di Dio e Ruggi d’Aragona, Via San Leonardo, 84131 Salerno, Italy; 4 Dipartimento di Medicina, Chirurgia e Odontoiatria, Università degli Studi di Salerno, Via S. Allende, 84081 Baronissi, Italy; fdalpiaz@unisa.it; 5 Azienda Ospedaliera di Perugia, 06132 Perugia, Italy; eleonoradistrutti@gmail.com

**Keywords:** gastric cancer, cancer cachexia, leukemia inhibitory factor (LIF), growth differentiation factor 15 (GDF15), bile acids, skeletal muscle wasting, metabolic remodeling

## Abstract

Cancer cachexia is a multifactorial metabolic syndrome characterized by progressive skeletal muscle and adipose tissue loss, systemic inflammation, and poor clinical outcomes, and represents a major unmet clinical need in gastric cancer. Growth Differentiation Factor 15 (GDF15) is a key mediator of cachexia-associated anorexia and tissue wasting; however, the upstream mechanisms regulating its expression in gastric cancer remain poorly defined. Leukemia Inhibitory Factor (LIF), a pleiotropic cytokine implicated in tumor progression and metabolic dysregulation, has emerged as a potential regulator of cachexia-related pathways. Here, we investigated the association between LIF in regulating GDF15 expression and its relationship with metabolic, inflammatory, and body composition alterations in gastric cancer. Transcriptomic profiling of paired neoplastic and non-neoplastic gastric mucosa from 61 gastric cancer patients revealed a significant upregulation of both LIF and GDF15 in tumor tissue, with a strong positive correlation between their expression levels. High GDF15 expression was associated with reduced overall survival, a finding validated in independent TCGA-STAD and ACRG cohorts. Intratumoral bile acid profiling uncovered a marked enrichment of primary bile acids and a depletion of secondary bile acids, resulting in reduced levels of bile acids with endogenous LIF receptor (LIFR) antagonist activity; elevated primary, LIFR non-antagonist bile acids were associated with worse survival outcomes. Clinically, increased LIF and GDF15 expression correlated with weight loss, heightened inflammatory burden, reduced serum protein and albumin levels, and impaired body composition in a sub-cohort of 19 patients. Notably, LIF expression showed a significant inverse association with both lumbar skeletal muscle index (L3SMI) and subcutaneous adipose tissue index (SATI). Mechanistically, experimental models demonstrated that LIF enhances proliferative activity in gastric cancer spheroids and exerts paracrine effects that impair myogenic differentiation and suppress hepatic metabolic gene expression. Collectively, these findings identify the LIF/GDF15 axis as a central driver of cancer-associated cachexia in gastric cancer and highlight LIF signaling as a potential therapeutic target.

## 1. Introduction

Cancer cachexia is a multifactorial metabolic syndrome characterized by involuntary weight loss, progressive depletion of skeletal muscle mass with or without loss of adipose tissue, and systemic inflammation [1]. It affects up to 80% of patients with advanced malignancies and represents a major determinant of poor prognosis, reduced tolerance to anticancer therapies, and impaired quality of life [2]. Among solid tumors, gastric cancer (GC) is particularly associated with cachexia due to the combined effects of tumor burden, gastrointestinal dysfunction, systemic inflammation, and metabolic derangements [3]. Despite its profound clinical impact, cancer cachexia remains largely refractory to current therapeutic strategies, underscoring the need to identify molecular drivers linking tumor biology to host metabolic alterations [4].

In recent years, Growth Differentiation Factor 15 (GDF15), a member of the transforming growth factor-β superfamily, has emerged as a central mediator of cancer-associated cachexia [5]. Elevated levels of GDF15 are consistently detected in oncology patients and correlate with weight loss, anorexia, muscle wasting, and poor survival [6,7]. Mechanistically, GDF15 acts through the GDNF family receptor α-like (GFRAL) receptor, localized in the brainstem to suppress appetite and induce aversive feeding behavior, while also exerting peripheral effects on lipid and muscle metabolism [8,9,10]. Preclinical studies have demonstrated that pharmacological blockade of the GDF15-GFRAL axis reverses anorexia and muscle wasting, highlighting its causal role in cachexia’s pathogenesis [11]. However, the upstream signals regulating GDF15 expression within the tumor microenvironment in gastric cancers remain incompletely understood [8].

The Leukemia Inhibitory Factor (LIF) is a pleiotropic cytokine belonging to the interleukin (IL)-6 family that signals through a heterodimeric receptor complex composed of LIF receptor (LIFR) and gp130, leading to activation of JAK/STAT, MAPK [12], HIPPO-YAP [13] and PI3K pathways [14]. Beyond its established roles in development and inflammation, LIF has gained increasing attention in oncology as a regulator of tumor progression, immune modulation, and metabolic reprogramming. LIF has been implicated in cancer cachexia through its ability to promote lipolysis and impaired anabolic signaling in skeletal muscle, while LIF administration promotes muscle atrophy in experimental models [15]. Elevated LIF levels have been reported in cachectic patients and are associated with systemic inflammation and muscle wasting, suggesting a role for LIF as a tumor-derived pro-cachectic mediator, linking cancer progression to host metabolic dysfunction [15,16].

Emerging evidence suggests a functional interplay between LIF and GDF15 in the regulation of energy homeostasis and catabolic responses [17,18,19]. Both cytokines are induced under stress and inflammatory conditions, share downstream signaling pathways, and have been independently implicated in anorexia and tissue wasting [20]. However, whether LIF acts as an upstream regulator of GDF15 expression in human cancers, and how this potential axis contributes to cachexia in gastric cancer, has not yet been elucidated. In parallel, increasing attention has been directed toward metabolic cues within the tumor microenvironment that may modulate cytokine signaling [21]. In this context, bile acids have recently been recognized not only as metabolic regulators but also as signaling molecules capable of modulating oncogenic pathways [22]. Notably, specific bile acids have been identified as endogenous antagonists of LIFR, providing a previously unrecognized mechanism by which metabolic alterations may influence LIF-driven signaling in cancer [23].

Here, we hypothesized that dysregulated LIF signaling in gastric cancer is associated with increased GDF15 expression and contributes to a coordinated cytokine network linked to cancer-associated cachexia.

To test this hypothesis, we have carried out an integrated analysis combining transcriptomic profiling of gastric cancer tissues, survival analyses in two independent patient cohorts, along with a detailed clinical and radiological assessment of cachexia-related features. Our results suggest that a LIF/GDF15 axis may contribute to cachexia development in gastric cancer patients and might be a potential target for therapeutic intervention [24].

## 2. Materials and Methods

### 2.1. Human Sample

#### 2.1.1. Human Internal Cohort

Gastric tissue samples were obtained from 61 patients with histologically confirmed gastric cancer who underwent curative surgical resection at the Surgical Unit of the University Hospital of Perugia (Italy). In the majority of cases (*n* = 56), paired specimens consisting of tumor tissue and adjacent non-neoplastic gastric mucosa were collected. Non-neoplastic samples were excised from areas macroscopically distant from the tumor to minimize the risk of microscopic tumor contamination. In addition, three tumor-only samples and two non-neoplastic-only specimens were included in the analysis. None of the enrolled patients had received neoadjuvant chemotherapy or radiotherapy prior to surgery. All participants provided written informed consent before sample collection. Tissue harvesting was performed intraoperatively by specialized personnel. Immediately after excision, specimens were placed in RNAlater stabilization solution, transported to the Gastroenterology Research Laboratory, and stored at −80 °C until further molecular processing. The study was conducted in compliance with ethical principles for biomedical research involving human subjects and was approved by the Ethics Committee of Regione Umbria (CEAS; permit FI00001, protocol no. 2266/2014) and by the Bioethics Committee of the University of Perugia (permits FIO0003, protocol no. 120687/2021 and FIO0009, protocol no. 357803/2024). All procedures adhered to institutional regulations and international ethical guidelines.

#### 2.1.2. Public Transcriptomic Datasets

Transcriptomic data from independent gastric cancer cohorts were retrieved from publicly available repositories. The GSE66229 dataset, generated by the Asian Cancer Research Group (ACRG), includes gene expression profiles from 300 gastric cancer samples and 100 non-neoplastic gastric tissues.

In addition, RNA-sequencing data from the Stomach Adenocarcinoma cohort of The Cancer Genome Atlas (TCGA-STAD) were analyzed, comprising 350 gastric cancer specimens and 31 normal gastric tissue samples. These external datasets were used for validation of gene expression patterns and survival analyses.

### 2.2. RNA Extraction, Library Preparation, and Transcriptomic Analysis

Total RNA was isolated from neoplastic and matched non-neoplastic gastric mucosa using the PureLink™ RNA Mini Kit (Thermo Fisher Scientific, Waltham, MA, USA), following the manufacturer’s protocol. RNA quantity and quality were evaluated using the Qubit™ RNA High Sensitivity Assay on a Qubit 3.0 fluorometer (Thermo Fisher Scientific), and RNA integrity was additionally verified by agarose gel electrophoresis. Transcriptome profiling libraries were generated using the Ion AmpliSeq™ Transcriptome Human Gene Expression Core Panel combined with the Chef-Ready Kit (Thermo Fisher Scientific, Waltham, MA, USA). Briefly, 10 ng of total RNA were reverse-transcribed into cDNA using the SuperScript™ VILO™ cDNA Synthesis Kit. Library construction, including barcoding and amplification, was performed in an automated manner on the Ion Chef™ System using the Ion Code™ PCR Plate, according to the manufacturer’s instructions. Prepared libraries were quantified, pooled at equimolar concentrations (final concentration 100 pM), and subjected to template preparation using the Ion 540™ Kit-Chef to generate template-positive Ion Sphere™ particles. Sequencing was carried out on the Ion S5™ sequencing platform (Thermo Fisher Scientific, Waltham, MA, USA). Raw sequencing data were processed using Torrent Suite™ Software (version 6). Differential gene expression analysis was performed using Transcriptome Analysis Console Software (version 4.0.2). Genes with a *p* value < 0.05 and an absolute fold change greater than 2 were considered significantly differentially expressed. The transcriptomic data obtained from the experimental cohort are publicly available in Mendeley Data, V1, doi: 10.17632/j46b34szhw.1.

### 2.3. Bile Acid Determinations

Gastric cancer samples were preserved at −80 °C and lyophilized. Then, 20 mg of each sample was manually homogenized using a potter pestle and dissolved in CH_3_OH at a final concentration 100 µg/µL for an opportune extraction for 2 h. Finally, they were diluted 5 times in a solution made of 50% H_2_O/50% MeOH, 0.1% formic acid (FA) and 5 mM ammonium acetate (AmAc).

Stock solutions of the individual BAs were distinctly prepared in MeOH, mixed and diluted in 50% H_2_O/50% MeOH, 5 mM AmAc and 0.1% FA to obtain the calibration standards ranging from 25 nM to 400 nM.

UHPLC-MRM-MS analyses were performed both on a QTRAP 6500 (AB Sciex) equipped Shimadzu Nexera LC and Auto Sampler systems and on a Xevo TQS Micro (Waters) equipped with an Acquity UPLC H-class chromatographer. In both sets, BAs mixture was separated on a Luna Omega 1.6 μm Polar (C18, 100 Å, 50 × 2.1 mm; Phenomenex) at 40 °C, and at a flow rate of 400 μL/min. For QTRAP 6500 set, the experimental conditions were reported in [25]. For Xevo TQS Micro set, the mobile phase A was H_2_O, 5 mM AmAc, 0.01% FA, and mobile phase B was MeOH/ACN (80:20), 5 mM AmAc, 0.01% FA. The gradient started at 50% B, increased to 55% B in 5 min and then to 70% B in 2 min and to 75% B in other 3 min, then was kept at 95% B for 5 min. The mass spectrometer was operated in negative MRM scanning mode using low collision energy to not fragment BAs, with the following parameters: cone potential at −50 V, collision energy at −5V and cell exit potential (CXP) at 20 V. Cone gas was set at 15 L/min, ion source gas at 300 L/min and ion spray voltage at −2500. Chromatograms were analyzed through the Quanlynx tool of the Masslynx software 4.2 (Waters) and through Analyst software 1.6.2 (AbSciex). 32 BAs have been quantified through the opportune standards curves.

### 2.4. Kaplan Mayer Analysis

Survival analyses were conducted using the Kaplan–Meier Plotter (KM Plotter) online tool accessed on September 2025 [26], which allows assessment of the prognostic impact of gene expression across large clinical cohorts. Gene expression data and corresponding survival information from our institutional cohort, together with publicly available datasets including TCGA-STAD and the GSE66229 ACRG cohort, were integrated into the platform for analysis. For patient stratification, the Auto-select best cutoff option [27] was employed to determine the optimal expression threshold for dichotomizing samples into high and low expression groups, this approach tests multiple expression thresholds and applies false discovery rate correction, although the selection of an optimized, data-driven cutoff may introduce bias and potentially overestimate prognostic effects. Kaplan–Meier survival curves were subsequently generated based on these groupings and exported for graphical representation and downstream evaluation.

### 2.5. L3SMI and SATI Score

Body composition analysis was performed in a sub-cohort of 19 gastric cancer patients with available preoperative computed tomography (CT) scans. Axial CT images acquired within 30 days prior to surgery were analyzed at the level of the third lumbar vertebra (L3), a validated anatomical landmark for the assessment of whole-body muscle and adipose tissue mass.

Skeletal muscle area was quantified by segmenting all muscle groups visible at L3 using predefined Hounsfield unit (HU) thresholds (−29 to +150 HU). The lumbar skeletal muscle index (L3SMI) was calculated by normalizing muscle cross-sectional area (cm^2^) to patient height squared (m^2^) and expressed as cm^2^/m^2^. Subcutaneous adipose tissue was segmented using standard HU thresholds for adipose tissue (−190 to −30 HU), and the subcutaneous adipose tissue index (SATI) was similarly normalized to height squared (cm^2^/m^2^), as previously described [24].

All measurements were performed blinded to clinical and molecular data. L3SMI and SATI were analyzed as continuous variables.

### 2.6. Cell Cultures

#### 2.6.1. Human Gastric Adenocarcinoma Cell Line (MKN45)

MKN45 were purchased from Japanese Collection of Research Bioresources (Human Science Research Resources Bank, Osaka, Japan). They were maintained in RPMi-1640 medium (Euroclone, reference ECB9006L), supplemented with 10% fetal bovine serum (FBS; Euroclone, Cat. No. ECS5000L), 1% L-glutamine (Euroclone, Cat. No. ECB3000D), and 1% penicillin/streptomycin (Euroclone, Cat. No. ECB30001D).

MKN45 gastric cancer cells were embedded in Cultrex UltiMatrix Reduced Growth Factor Basement Membrane Extract (Cat. No. BME001-05) at a density of 1 × 10^4^ cells per well in 24-well plates and allowed to form three-dimensional spheroids. Spheroids were cultured until day 4, after which they were serum-starved for 24 h and subsequently left untreated or stimulated with recombinant human LIF (10 ng/mL). Cultures were then maintained until day 8. At the end of the experiment, culture supernatants were collected, clarified by centrifugation, and used as conditioned media for the experiments described below, while spheroids were processed for immunofluorescence analysis.

#### 2.6.2. Human Liver Cell Line Derived from a Hepatocarcinoma (HepaRG)

HepaRG cells (Life Technologies Italia, Monza, Italy) were cultured in William’s E medium without phenol red (Thermo Fisher Scientific, Waltham, MA, USA, A1217601), supplemented with maintenance additives (Thermo Fisher Scientific, Waltham, MA, USA, CM4000) and 10% fetal bovine serum (FBS). Cells were seeded at a density of 2 × 10^5^ cells per well. After 24 h, cultures were serum-starved for 1 h and subsequently exposed to conditioned medium derived from MKN45 spheroids, either untreated or stimulated with LIF, as described above. Conditioned media were diluted 1:2 with HepaRG culture medium and applied for 24 h. At the end of the stimulation period, HepaRG cells were harvested and analyzed by quantitative PCR (qPCR).

#### 2.6.3. Myoblast Cell Line (C2C12)

C2C12 murine myoblasts were obtained from ATCC (Manassas, VA, USA) and maintained in Dulbecco’s Modified Eagle Medium (DMEM; Euroclone, Cat. No. ECB7501L) supplemented with 20% fetal bovine serum (FBS), 1% L-glutamine (Euroclone, Cat. No. ECB3000D), and 1% penicillin/streptomycin (Euroclone, Cat. No. ECB30001D). Cells were seeded at a density of 2 × 10^5^ cells per well in 6-well plates. Myogenic differentiation was initiated 24 h after seeding by switching to differentiation medium consisting of DMEM supplemented with 2% horse serum. Where indicated, differentiation was carried out either under standard conditions or in the presence of conditioned medium derived from MKN45 spheroids, untreated or stimulated with LIF, diluted 1:2 in differentiation medium. Differentiation medium was refreshed on day 5. On day 6, myotube formation was documented by phase-contrast microscopy. Cross-sectional diameters of differentiated myotubes were quantified as described below, and cells were subsequently processed for immunofluorescence analysis.

### 2.7. Quantification of Myotube Cross-Sectional Diameter

Myotube morphology was assessed by phase-contrast microscopy using an inverted microscope under identical magnification and exposure settings. Images were acquired using an inverted microscope under identical magnification and exposure settings for all experimental conditions. For each condition, multiple random fields were captured from at least three independent wells to ensure representative sampling. Cross-sectional diameters of myotubes were measured using ImageJ software version 1.53 (NIH, Betheseda, MD, USA). Individual myotubes were identified based on elongated morphology and multinucleation. For each myotube, diameter was measured at three distinct points along the longitudinal axis, avoiding branching regions, and the mean value was calculated. All measurements were performed in a blinded manner. Data are expressed as mean ± SEM and were used for subsequent statistical analyses.

### 2.8. Immunofluorescence Analysis

Immunofluorescence analyses were performed on three-dimensional spheroids derived from MKN45 gastric cancer cells and on differentiated C2C12 myotubes. Samples were fixed in 4% paraformaldehyde for 15 min at room temperature, washed in phosphate-buffered saline (PBS), and permeabilized with 0.1% Triton X-100 in PBS for 10 min. Non-specific binding was blocked by incubation with blocking solution consisting of PBS supplemented with 10% horse serum and 1% bovine serum albumin (BSA) for 1 h at room temperature. Samples were incubated overnight at 4 °C with the appropriate primary antibodies diluted in blocking buffer, as detailed below. The following day, samples were washed three times with PBS containing 0.1% Tween-20 (PBST) and incubated with fluorophore-conjugated secondary antibodies for 1 h at room temperature in the dark. To reduce autofluorescence, samples were treated with Sudan Black for 5 min, followed by extensive washing in PBS. Nuclei were counterstained with DAPI for 1 min at room temperature in the dark. After final washes, samples were mounted using ProLong™ Glass Antifade Mountant (Thermo Fisher Scientific, Waltham, MA, USA), sealed, and analyzed using a fluorescence microscope (Olympus BX60). Image acquisition was performed using identical exposure and acquisition settings for all experimental conditions.

A complete list of primary and secondary antibodies used is provided in the Table 1.

### 2.9. Quantification of αSMA Immunofluorescence

Quantitative analysis of αSMA immunofluorescence was performed using ImageJ software version 1.53 (NIH, Bethesda, MD, USA). For each experimental condition, fluorescence images were acquired using identical microscope settings (exposure time, gain, and magnification) to allow direct comparison between groups. Regions of interest (ROIs) were manually defined around individual myotubes based on morphology and αSMA-positive signal. For each ROI, the mean fluorescence intensity was measured. Background fluorescence was determined by selecting adjacent areas devoid of specific staining within the same field of view. Corrected fluorescence intensity was calculated by subtracting the mean background signal from the mean ROI value.

At least five independent fields per well and five samples per condition were analyzed. All measurements were performed in a blinded manner. Data are expressed as mean ± SEM and were used for subsequent statistical analyses.

### 2.10. Reverse Transcription e qPCR

Total RNA was extracted from HepaRG cells using the Direct-zol™ RNA MicroPrep Kit equipped with Zymo-Spin™ IIC Columns (Zymo Research, Irvine, CA, USA), in accordance with the manufacturer’s instructions. The isolated RNA was then used for quantitative PCR (qPCR) experiments.

Residual genomic DNA was removed by DNase I treatment (Thermo Fisher Scientific, Waltham, MA, USA). Subsequently, 1 μg of RNA was reverse-transcribed into cDNA using SuperScript™ II Reverse Transcriptase (Thermo Fisher Scientific, Waltham, MA, USA) and random hexamer primers, in a final reaction volume of 20 μL.

Quantitative PCR was carried out using 10 ng of cDNA in a total volume of 20 μL, containing 200 nM of each primer and 10 μL of SYBR™ Select Master Mix (Thermo Fisher Scientific, Waltham, MA, USA).

All qPCR reactions were performed in technical triplicate using a StepOnePlus™ Real-Time PCR System (Applied Biosystems, Foster City, CA, USA). The thermal cycling protocol consisted of an initial denaturation step at 95 °C for 3 min, followed by 40 cycles of amplification at 95 °C for 15 s, 56 °C for 20 s, and 72 °C for 30 s.

Relative gene expression levels were determined using the comparative ΔCt method. Primer pairs were designed with PRIMER3 software v.0.4.0 (http://bioinfo.ut.ee/primer3-0.4.0/) accessed on 15 February 2026 based on reference sequences obtained from the NCBI database. The primer sequences used for mouse genes (forward and reverse) are listed in Table 2.

### 2.11. Protein Extraction and Western Blot Analysis

MKN45 gastric cancer cells were seeded at 1.5 × 10^6^ cells/well and cultured for 24 h. Then, cells were washed with cold phosphate-buffered saline and lysed in RIPA buffer supplemented with protease and phosphatase inhibitor cocktails. Whole-cell lysates were clarified by centrifugation, and protein concentrations were determined prior to electrophoretic separation. Equal amounts of protein were resolved on 10% Tris–glycine polyacrylamide gels (Invitrogen) and transferred onto nitrocellulose membranes using the iBlot™ 2 Dry Blotting System (Invitrogen, Carlsbad, CA, USA). Membranes were blocked for 1 h at room temperature in Tris-buffered saline containing 0.1% Tween-20 (TBS-T) and 5% non-fat dry milk. Membranes were incubated overnight at 4 °C with primary antibodies against Vimentin (NBP1-926871:1000), LIF (PA5-79600, 1:500) and LIFR (ab235908 1:500).

### 2.12. LIF Quantification by ELISA

LIF concentrations in conditioned media were quantified using a human LIF ELISA kit (Antibodies.com, Cambridge, UK, catalog number A2990), according to the manufacturer’s instructions. Briefly, conditioned media were collected from MKN45 gastric cancer spheroids left untreated or stimulated with recombinant human LIF (10 ng/mL) for 24 h. Supernatants were clarified by centrifugation to remove cellular debris and stored at −80 °C until analysis. Samples and standards were added to ELISA plates and incubated as specified by the kit protocol. Absorbance was measured using a microplate reader at the recommended wavelength, and LIF concentrations were calculated by interpolation from a standard curve generated using recombinant human LIF provided with the kit. Results are expressed as pg/mL and represent the mean ± SEM of independent experiments.

### 2.13. Statistical Analysis

We first performed the Kolmogorov–Smirnov test to assess whether our data are in a normal distribution. The Student t test was performed on an experimental set composed of two groups: Welch’s correction for samples with Gaussian distribution and Mann–Whitney test for data without a Gaussian distribution. For the correlation studies, the correlation was calculated with Pearson r for data with Gaussian distribution and with Spearman r for data that did not have a Gaussian distribution. All test was carried out using the Prism 8.0 software (GraphPad) (* *p* < 0.05).

## 3. Results

LIF overexpression correlates with GDF15 expression and predicts poor prognosis in neoplastic gastric mucosa.

**Figure 1:**
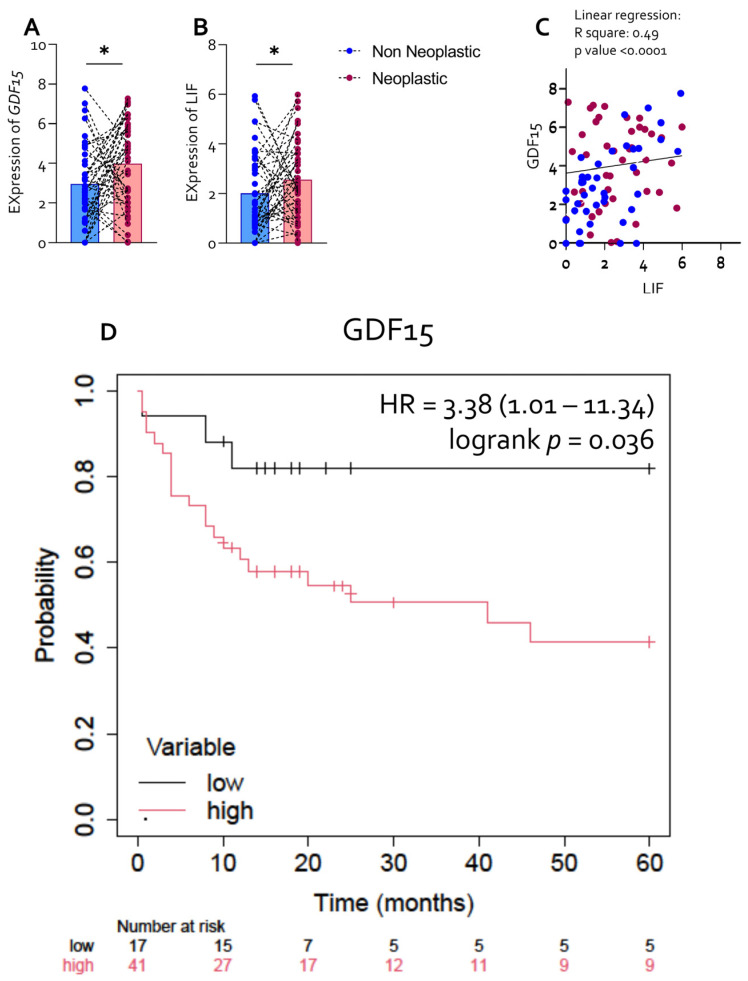
**LIF Overexpression correlates with GDF15 expression and predicts a poor prognosis in Neoplastic GC Mucosa.** Transcriptome analysis of non-neoplastic and neoplastic tissues was carried out in 61 GC patients, who underwent surgical resection at Perugia University Hospital. Gene expression (Log2) of: (**A**) LIF and (**B**) GDF15. (**C**) Correlation analysis between LIF and GDF15. Kaplan Meyer analysis of (**D**) GDF15. Each dot represents a patient. Results are the mean ± SEM. * *p* < 0.05.

Transcriptomic profiling was performed on paired non-neoplastic and neoplastic gastric mucosa obtained from 61 patients with gastric cancer (GC) who underwent surgical resection at Perugia University Hospital (Table 3). Gene expression analysis revealed a significant upregulation of both *LIF* and *GDF15* in neoplastic tissues compared with matched non-neoplastic counterparts (Figure 1A,B).

Each data point represents an individual patient, and results are expressed as mean ± SEM. Correlation analysis demonstrated a strong positive association between *LIF* and *GDF15* expression levels across the analyzed samples (R^2^ = 0.49, *p* < 0.0001; Figure 1C), indicating coordinated expression of these two cachexia-related factors in gastric cancer tissue. To assess the clinical relevance of *GDF15* expression, patients were stratified according to high or low tumor *GDF15* levels. Kaplan–Meier survival analysis revealed that elevated *GDF15* expression was significantly associated with reduced overall survival (HR = 3.38, 95% CI 1.01–11.34; log-rank *p* = 0.036; Figure 1D), identifying *GDF15* as a negative prognostic marker in this cohort. Similarly, Kaplan–Meier analysis based on tumor LIF expression suggested a trend toward poorer overall survival at higher expression levels (Supplementary Figure S1).

**Figure 2:**
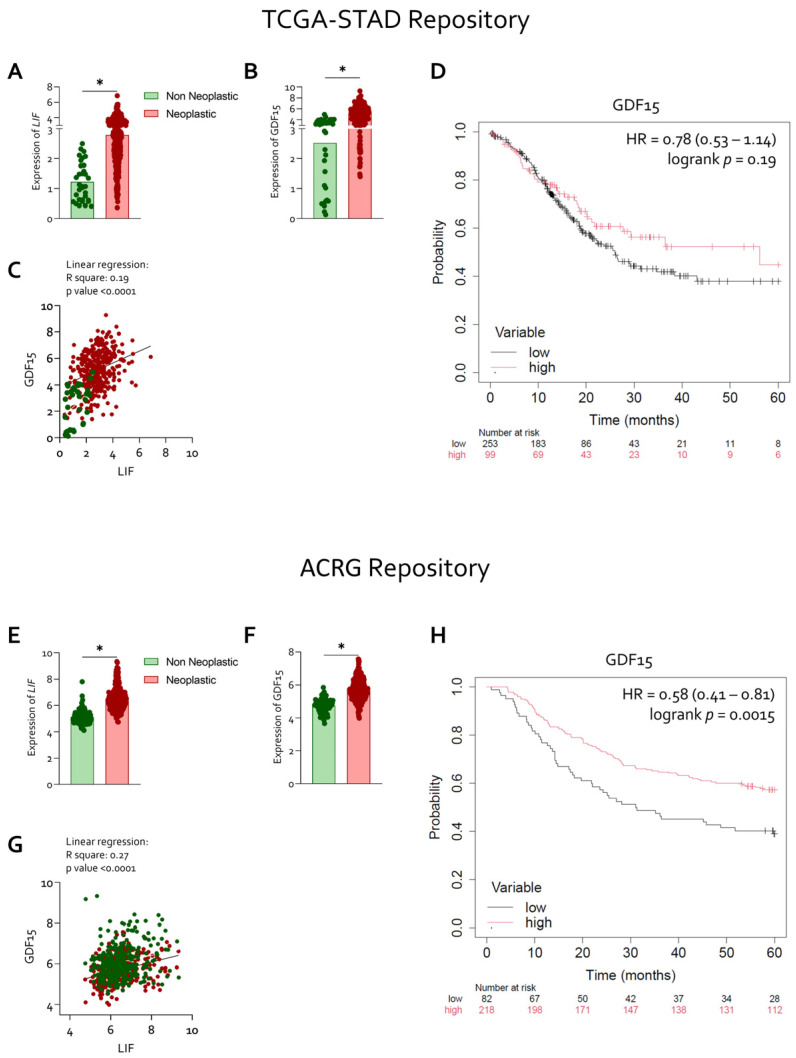
**Overexpression of LIF parallels GDF15 upregulation in neoplastic gastric mucosa and serves as a marker of adverse clinical outcome.** RNA-seq analysis of healthy, neoplastic mucosa, from TGCA-STAD repository and ACRG was reported. Gene expression (Log2) of: (**A**–**E**) LIF and (**B**–**F**) GDF15. (**C**–**G**) Correlation analysis between LIF and GDF15. Kaplan Meyer analysis of (**D**–**H**) GDF15. Each dot represents a patient. Results are the mean ± SEM. * *p* < 0.05.

These findings were independently validated using publicly available RNA-seq datasets from the TCGA-STAD and ACRG repositories. In both cohorts, *LIF* and *GDF15* expression was significantly higher in neoplastic gastric mucosa compared with non-neoplastic tissue (Figure 2A,B,E,F).

Consistent with our internal cohort, correlation analyses confirmed a positive association between *LIF* and *GDF15* expression in both TCGA (R^2^ = 0.19, *p* < 0.0001; Figure 2C) and ACRG datasets (R^2^ = 0.27, *p* < 0.0001; Figure 2G). Survival analyses further demonstrated that high *GDF15* expression was associated with worse overall survival in the ACRG cohort (HR = 0.58, 95% CI 0.41–0.81; log-rank *p* = 0.0015; Figure 2H), whereas no significant survival difference was observed in the TCGA cohort (Figure 2D). Differences in patient composition, disease stage, and data acquisition platforms between cohorts may contribute to these discrepant survival associations.

Collectively, these results demonstrate that *LIF* and *GDF15* are concomitantly overexpressed in gastric cancer tissue and that elevated *GDF15* levels are associated with adverse clinical outcomes across independent patient cohorts. While these data indicate coordinated expression of LIF and GDF15 in gastric cancer tissue, they do not define a causal hierarchy between the two factors.

### 3.1. Secondary Bile Acids Acting as Endogenous LIFR Antagonists Are Reduced in Neoplastic Gastric Mucosa

**Figure 3:**
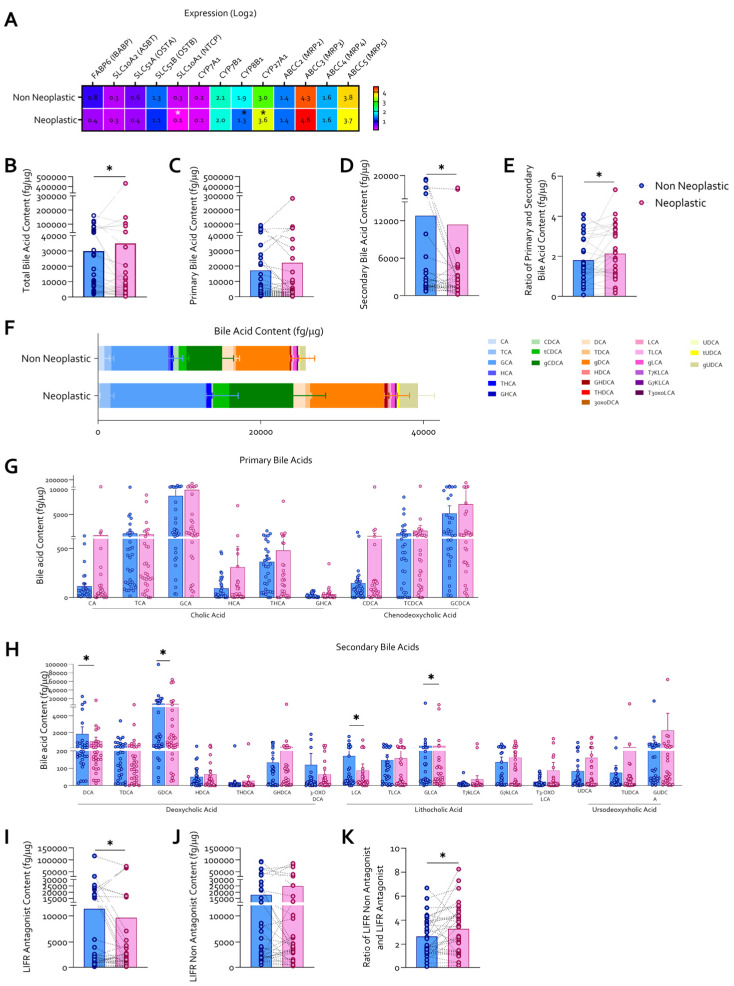
**Secondary bile acids, endogenous LIFR antagonists, are reduced in neoplastic gastric mucosa.** (**A**) Heatmap of gene expression (log_2_) involved in bile acid homeostasis. (**B**) Total bile acid content (fg/μg). Content of (**C**) primary bile acids (fg/μg), (**D**) secondary bile acids (fg/μg), and (**E**) their relative ratios in the two tissue compartments. (**F**) Representative histogram of bile acid composition in neoplastic and non-neoplastic mucosa (fg/μg). (**G**) Levels of individual primary bile acids and their conjugated derivatives (fg/μg). (**H**) Content of secondary bile acids and their conjugated derivatives (fg/μg). Content of (**I**) LIFR Antagonist Bile Acids (fg/μg); (**J**) LIFR Non-Antagonist (fg/μg) and (**K**) Ratio between LIFR Antagonist and Non-Antagonist Bile Acids. * *p* < 0.05.

To investigate alterations in bile acid metabolism within the gastric tumor microenvironment, bile acid profiling was performed in a subset of 32 paired non-neoplastic and neoplastic gastric mucosa samples from patients with gastric cancer. Transcriptomic analysis of genes involved in bile acid synthesis, transport, and signaling revealed marked differences between the two tissue compartments (Figure 3A), characterized by a reduction in NTCP (SLC10A1) and CYP8B1 expression and a concomitant increase in CYP27A1 in neoplastic mucosa.

These transcriptional changes were detected in bulk tumor tissue and are consistent with a shift toward altered bile acid uptake and synthesis pathways within the neoplastic gastric mucosa.

Quantitative mass spectrometry analysis demonstrated that total bile acid content was significantly increased in neoplastic gastric mucosa compared with non-neoplastic tissue (Figure 3B). Primary bile acids were significantly enriched in tumor samples (Figure 3C), whereas secondary bile acids were markedly reduced (Figure 3D), resulting in a significantly increased primary-to-secondary bile acid ratio in neoplastic tissue (Figure 3E). A compositional analysis further highlighted distinct bile acid signatures between non-neoplastic and neoplastic mucosa (Figure 3F). Detailed quantification of individual bile acids revealed a trend toward increased levels of several primary bile acids and their conjugated derivatives, including cholic acid and chenodeoxycholic acid-derived species, in neoplastic samples (Figure 3G). In contrast, multiple secondary bile acids and their conjugated forms were significantly reduced in tumor tissue (Figure 3H), such as DCA, LCA and their Glyco-derivatives, previously demonstrated as potent inhibitors of LIFR [23].

Based on their previously characterized functional activity as endogenous antagonists of the LIFR, bile acids were further classified into LIFR antagonist and non-antagonist groups. Tumor tissue displayed a significant reduction in the total content of LIFR antagonist bile acids (Figure 3I), while levels of LIFR non-antagonist bile acids were relatively preserved or increased (Figure 3J). Consequently, the ratio between LIFR antagonist and non-antagonist bile acids was significantly decreased in neoplastic gastric mucosa compared with non-neoplastic tissue (Figure 3K). However, whether the intratumoral concentrations of these bile acids are sufficient to quantitatively modulate LIFR signaling in vivo remains to be determined.

**Figure 4:**
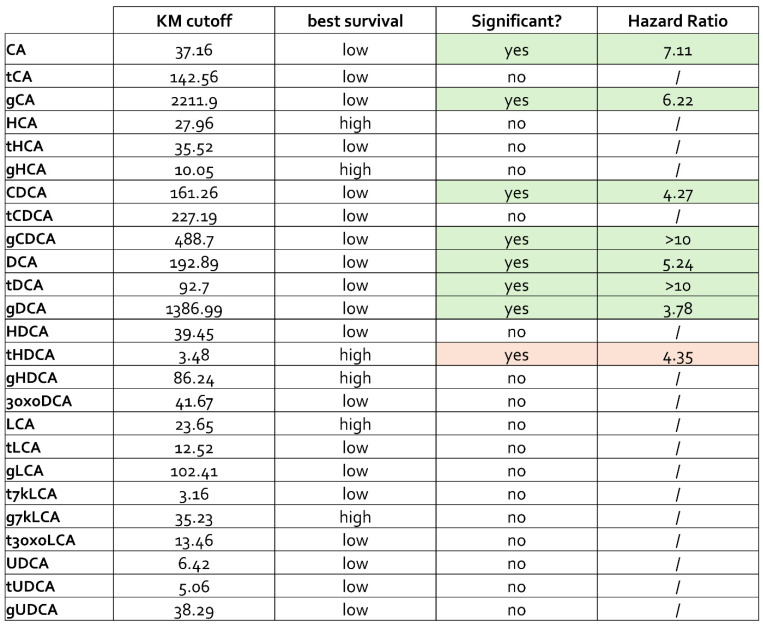
**Increased concentrations of primary, non-antagonist bile acids correlate with worse survival.** Kaplan–Meier survival analysis stratified by intratumoral bile acid levels in gastric cancer, highlighting the negative prognostic impact of elevated primary, LIFR non-antagonist bile acids. Green shading indicates bile acids associated with lower survival, whereas red shading indicates bile acids associated with higher survival.

To assess the clinical relevance of these metabolic alterations, Kaplan–Meier survival analyses were performed by stratifying patients according to intratumoral bile acid concentrations. Elevated levels of primary bile acids, which predominantly belong to the LIFR non-antagonist group, were significantly associated with worse overall survival (Figure 4).

In contrast, higher intratumoral concentrations of specific secondary bile acids, classified as LIFR antagonists, were associated with improved survival outcomes.

These findings support the notion that gastric cancer is characterized by a selective depletion of secondary bile acids with endogenous LIFR antagonist activity, alongside an accumulation of primary bile acids lacking such antagonistic properties, and that this imbalance is associated with adverse clinical outcomes.

### 3.2. High LIF and GDF15 Expression Associates with Impaired Nutritional Status and Increased Inflammatory Burden

**Figure 5:**
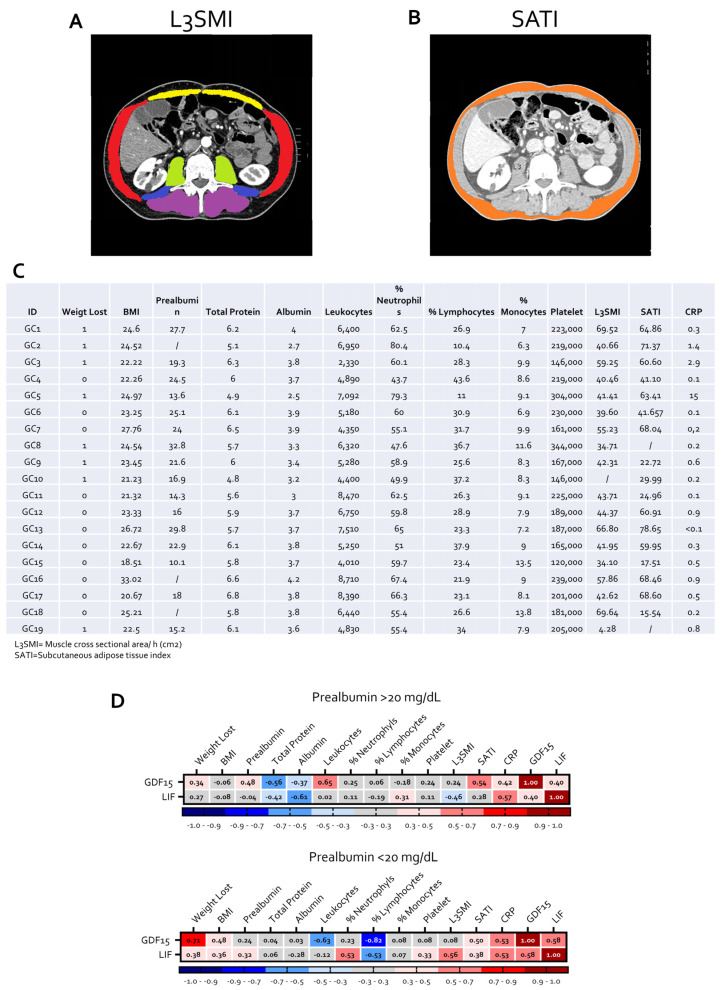
**High LIF and GDF15 expression associates with impaired nutritional status and increased inflammatory burden.** Representative CT images for the assessment of (**A**) skeletal muscle mass at the third lumbar vertebra (L3SMI)and (**B**) subcutaneous adipose tissue index (SATI)). In the CT images at the L3 level, skeletal muscles are color-segmented as follows: psoas (blue), paraspinal muscles (purple), quadratus lumborum (green), abdominal wall muscles (red), and rectus abdominis (yellow). Subcutaneous adipose tissue is displayed in orange (**C**) Table summarizing nutritional, inflammatory, and body composition parameters in gastric cancer patients. (**D**) Correlation matrices showing the relationships between clinical and nutritional variables, body composition parameters, inflammatory markers, and GDF15/LIF expression levels in gastric cancer patients stratified according to serum prealbumin levels. Red indicate a positive correlation and Blue a negative correlation. Left panel: patients with prealbumin ≥ 20 mg/dL. Right panel: patients with prealbumin < 20 mg/dL. Results are expressed as correlation coefficients.

To explore the relationship between LIF/GDF15 expression and systemic features of cancer-associated cachexia, nutritional status, body composition, and inflammatory parameters were evaluated in a cohort of gastric cancer patients. Skeletal muscle mass and adipose tissue distribution were assessed on computed tomography (CT) images at the level of the third lumbar vertebra (L3), allowing the calculation of the lumbar skeletal muscle index (L3SMI) and subcutaneous adipose tissue index (SATI), as illustrated in representative images (Figure 5A,B) [24].

Clinical, nutritional, inflammatory, and body composition parameters for individual patients are summarized in Figure 5C.

Correlation analyses were then performed to assess the association between circulating and tumor-related factors and markers of nutritional status and inflammation. Correlation matrix analysis revealed that both GDF15 and LIF expression levels were associated with multiple indicators of impaired nutritional status and systemic inflammation (Figure 5D). To improve interpretability, patients were stratified according to serum prealbumin levels (<20 vs. ≥20 mg/dL), a clinically validated threshold for nutritional risk [29]. In patients with prealbumin levels ≥20 mg/dL (Figure 5D, on left), GDF15 expression showed positive correlations with weight loss, SATI, leukocyte counts, C-reactive protein (CRP) levels, and LIF expression, while displaying inverse correlations with total protein and albumin levels. In the same subgroup, LIF expression was positively associated with CRP levels and monocyte percentages and inversely correlated with total protein, albumin and L3SMI. In contrast, in patients with prealbumin levels <20 mg/dL (Figure 5D, on right), these associations were more pronounced. In this subgroup, GDF15 expression showed stronger positive correlations with weight loss, body mass index reduction, SATI, CRP levels, and LIF expression, while inversely correlating with total protein and albumin levels. Notably, GDF15 also displayed inverse correlations with leukocyte and lymphocyte percentages, consistent with a more profound systemic inflammatory and immunosuppressive phenotype. Within the same subgroup, LIF expression positively correlated with CRP levels and weight loss and inversely correlated with leukocyte counts.

Overall, these findings indicate that the association between tumor LIF/GDF15 expression and cachexia-related features is more evident in patients with impaired nutritional status. Given the limited sample size and the cross-sectional nature of the analysis, these associations should be interpreted as correlative, and the study was not powered to perform multivariate analyses adjusting for potential confounders such as tumor stage, disease burden, or systemic inflammation.

**Figure 6:**
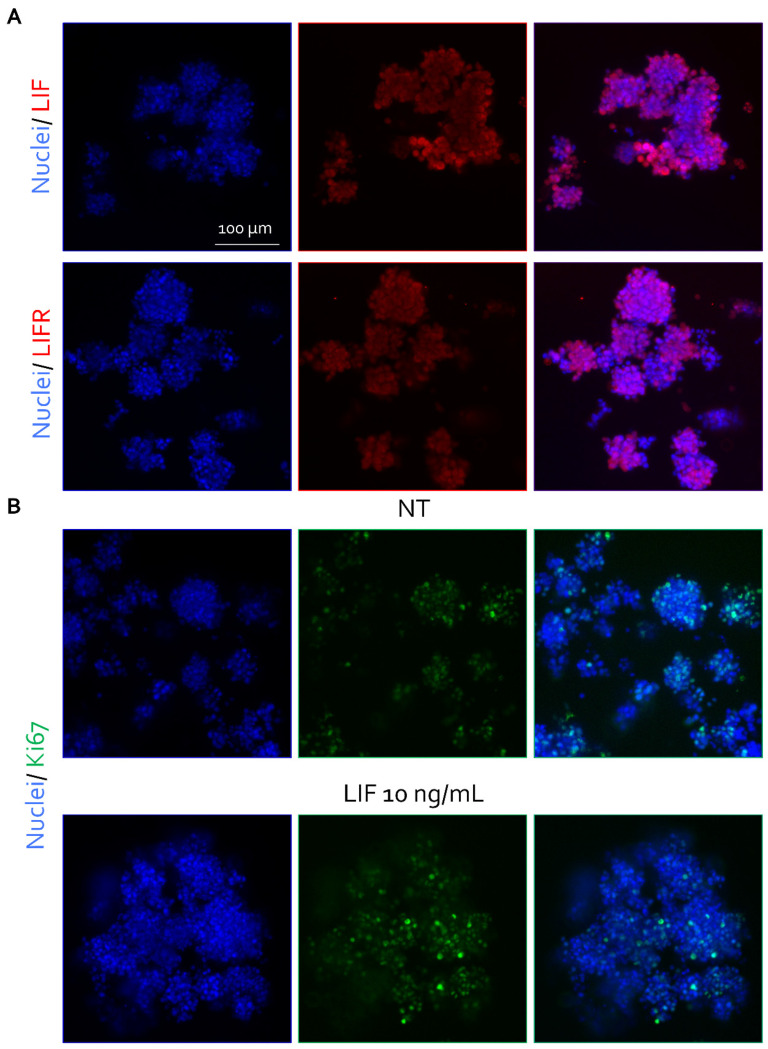
**LIF signaling increases proliferative activity, supporting an aggressive phenotype in gastric cancer spheroids.** (**A**) Representative immunofluorescence analysis showing basal expression of LIF and LIF receptor (LIFR) in untreated (NT) MKN45 three-dimensional spheroids. LIF and LIFR are shown in red. Nuclei were counterstained with DAPI. (**B**) Representative immunofluorescence staining of the proliferation marker Ki67 (green) in MKN45 spheroids left untreated (NT) or stimulated with recombinant human LIF (10 ng/mL). Nuclei were counterstained with DAPI.

To explore whether the association between elevated LIF/GDF15 expression and cachexia-related features reflected a functional tumor-driven mechanism, we next investigated the effects of LIF signaling in experimental models. Immunofluorescence and Western Blot analysis revealed that MKN45 gastric cancer spheroids basally expressed both LIF and its receptor LIFR, indicating the presence of an active autocrine signaling axis (Figure 6A and Supplementary Figures S2 and S3A).

**Figure 7:**
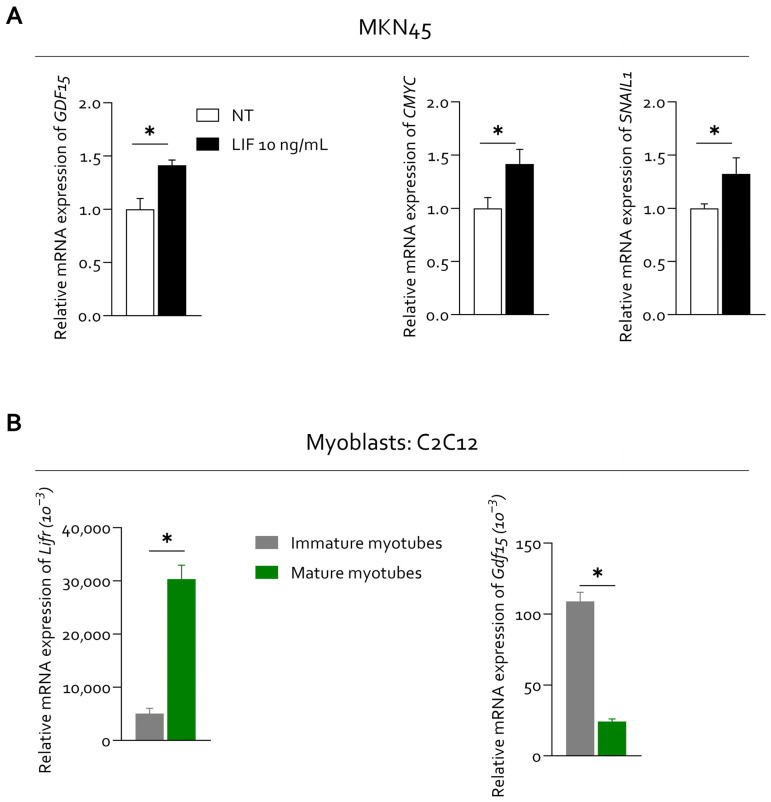
**LIF induces GDF15 expression in gastric cancer cells.** (**A**) Relative mRNA expression levels of *GDF15*, *CMYC* and *SNAIL1* (from left to right) were analyzed by quantitative PCR in MKN45 cells. Data are expressed as mean ± SEM (*n* = 3). Each value is normalized to *RPLP0* and is expressed relative to those of NT, which are arbitrarily set to 1. * *p* < 0.05. (**B**) Relative mRNA expression levels of LIFR and GDF15 in immature and mature myotubes. Data are expressed as mean ± SEM (*n* = 5) Each value is normalized to *Hprt1* and is expressed relative to those of NT, which are arbitrarily set to 1.

Exposure to recombinant human LIF (10 ng/mL) resulted in a marked increase in spheroid proliferative activity, as indicated by enhanced Ki67 staining, consistent with a more aggressive tumor phenotype (Figure 6B and Supplementary Figures S2 and S3B). At the transcriptional level, LIF stimulation induced the transcriptional upregulation of *GDF15*, together with the proliferation-associated gene *c-MYC* and the epithelial–mesenchymal transition marker *SNAIL1*, supporting the activation of tumor programs linked to aggressiveness and cachexia-related signaling (Figure 7A). Conversely, pharmacological inhibition of LIFR signaling, using LRI305 or the previously described Compound 1h [30], significantly attenuated the expression of *GDF15*, *KI-67* and *SNAIL1*, indicating that these transcriptional responses are dependent on LIFR activation (Supplementary Figure S4).

In parallel, gene expression analyses demonstrated that *LIFR* is expressed in C2C12 cells, and that *GDF15* expression is increased in immature C2C12 myoblasts, supporting the ability of skeletal muscle cells to respond to LIF-related signals (Figure 7B).

**Figure 8:**
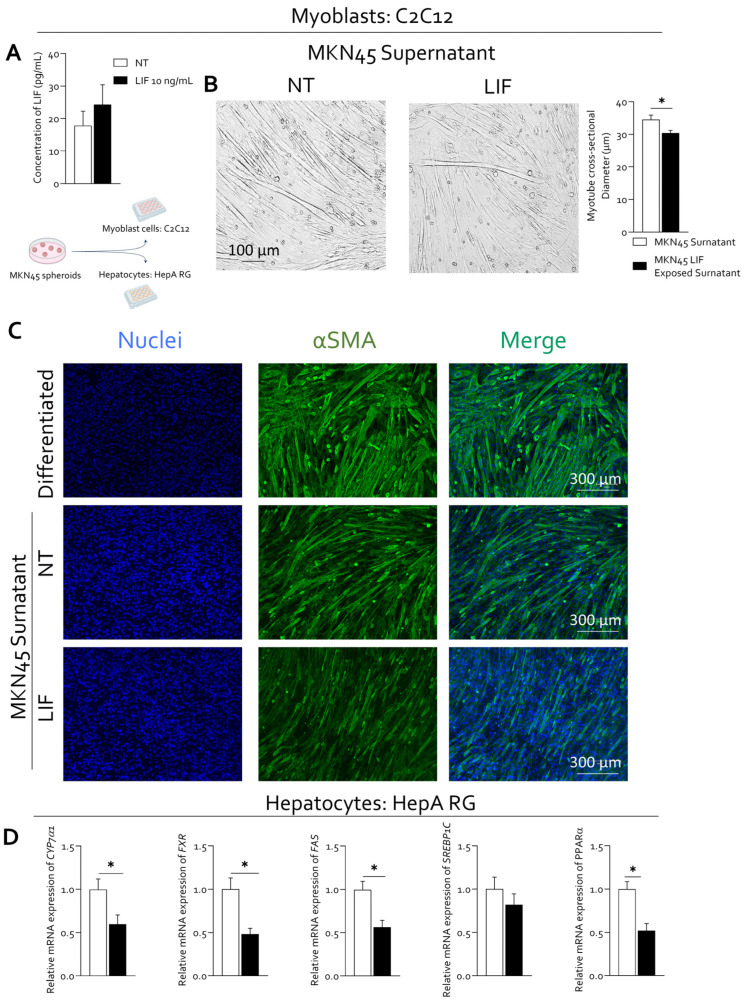
**Tumor-derived LIF signaling exerts paracrine effects on muscle and hepatic cells.** (**A**) Quantification of LIF concentrations in conditioned media from untreated (NT) and LIF-stimulated MKN45 spheroids measured by ELISA (up). Schematic representation of the experimental workflow. MKN45 spheroids were left untreated or stimulated with LIF, and conditioned media were subsequently collected and used to stimulate C2C12 myoblasts and HepaRG hepatocytes (down). (**B**) Representative phase-contrast images of differentiated C2C12 myotubes exposed to conditioned media derived from untreated or LIF-stimulated MKN45 spheroids. Quantification of myotube cross-sectional diameter is shown on the right. Data are expressed as mean ± SEM. * *p* < 0.05. (**C**) Immunofluorescence analysis of αSMA (green) in differentiated C2C12 myotubes and C2C12 myotubes exposed to conditioned media from untreated or LIF-stimulated (10 ng/mL) MKN45 spheroids). Nuclei were counterstained with DAPI. (**D**) Relative mRNA expression levels of *CYP7A1*, *FXR*, *FAS*, *SREBP1c*, and *PPARα* (from left to right) were analyzed by quantitative PCR in HepaRG cells. Data are expressed as mean ± SEM (*n* = 5). Each value is normalized to *RPLP0* and is expressed relative to those of NT, which are arbitrarily set to 1. * *p* < 0.05.

Conditioned media collected from untreated and LIF-stimulated MKN45 spheroids were then used to assess paracrine effects on skeletal muscle cells (Figure 8A). ELISA measurements revealed detectable basal levels of LIF in conditioned media from untreated spheroids (approximately 17 pg/mL), which were increased following LIF stimulation (approximately 24 pg/mL). While this increase may reflect both the presence of exogenously added LIF and/or enhanced endogenous LIF production, these data confirm that conditioned media derived from LIF-stimulated spheroids are enriched in LIF-related signaling (Figure 8A).

Differentiated C2C12 myotubes exposed to conditioned media derived from LIF-treated spheroids displayed a significant reduction in myotube cross-sectional diameter compared with those treated with conditioned media from untreated spheroids (Figure 8B).

This morphological alteration was accompanied by reduced expression of αSMA, as assessed by immunofluorescence analysis, consistent with altered cytoskeletal organization and compromised myogenic maturation in myotubes exposed to LIF-enriched paracrine conditions (Figure 8C). Notably, fully differentiated C2C12 myotubes cultured in standard differentiation medium exhibited markedly higher αSMA expression compared with both myotubes exposed to conditioned media from untreated MKN45 spheroids and those exposed to LIF-stimulated spheroid-derived media, indicating that tumor-derived paracrine signals suppress normal myogenic cytoskeletal maturation (Figure 8C).

In parallel, the impact of tumor-derived LIF signaling on hepatic metabolic pathways was evaluated using HepaRG cells. Exposure to conditioned media from LIF-stimulated MKN45 spheroids for 24 h resulted in a significant downregulation of genes involved in bile acid synthesis and lipid metabolism, including *CYP7A1*, *FXR*, *FAS*, *PPARα*, and *SREBP1c*, compared with cells treated with conditioned media from untreated spheroids (Figure 8D). These changes indicate a suppression of key metabolic regulatory pathways in hepatocyte-like cells in response to LIF-enriched tumor-derived signals.

Collectively, these experimental findings demonstrate that LIF signaling enhances gastric cancer spheroid proliferation and exerts deleterious paracrine effects on both skeletal muscle differentiation and hepatic metabolic gene expression, supporting a mechanistic link between tumor-derived LIF activity and the systemic metabolic alterations observed in gastric cancer-associated cachexia.

## 4. Discussion

This study provides a comprehensive clinical, transcriptomic, and metabolic characterization of the LIF-GDF15 axis in gastric cancer, highlighting its association with cancer progression and cachexia-related phenotypes. By integrating analyses of paired gastric tissues, large independent transcriptomic datasets, bile acid profiling, and detailed clinical-nutritional assessments, we identify LIF and GDF15 as two mediators at the intersection between tumor-intrinsic signaling and host metabolic derangement.

Our results demonstrate that in gastric cancers, both LIF and GDF15 are significantly overexpressed in neoplastic mucosa compared with matched non-neoplastic samples. This finding was consistently observed in our internal cohort as well and, independently validated by the TCGA and ACRG datasets, underscoring the robustness of LIF-GDF15 upregulation in gastric cancers. The strong positive correlation between LIF and GDF15 expression across three cohorts suggests a coordinated regulation of these factors within the tumor microenvironment. Importantly, elevated intratumoral GDF15 mRNA levels correlated with reduced overall survival, identifying GDF15 as a negative prognostic marker and supporting its relevance in gastric cancer progression [31]. Elevated GDF15 has been widely associated with poor outcomes and cachexia across multiple cancer types [32].

Beyond these prognostic associations, our findings provide evidence that expression of LIF-GDF15 is associated with markers of disease severity. Thus: (1) high expression levels of both factors correlated with impaired nutritional status; (2) reduced levels of nutrition biomarkers; and (3) decreased skeletal muscle mass, as assessed by CT-derived body composition parameters. These alterations are consistent with the hallmark features of cancer-associated cachexia, a multifactorial syndrome characterized by progressive weight loss, muscle wasting, and systemic inflammation [32]. The observed associations between LIF/GDF15 expression and inflammatory cell profiles further support a link between tumor-derived cytokines and systemic inflammatory burden, a central driver of cachexia pathophysiology [33].

A novel aspect of this study is the integration of bile acid metabolism into the regulation of LIF signaling in gastric cancer. Bile acid profiling revealed a profound remodeling of the intratumoral bile acid pool, characterized by an accumulation of primary bile acids and a concomitant depletion of secondary bile acids in neoplastic gastric mucosa. These metabolic changes were accompanied by transcriptional alteration in the expression of genes involved in bile acid transport and synthesis, including reduced expression of NTCP and CYP8B1 and increased expression of CYP27A1, indicative of altered bile acid uptake and synthetic pathways within bulk tumor tissue [34].

Strikingly, when bile acids were functionally classified according to their ability to antagonize the LIFR, we found that, in comparison to their non-neoplastic pairs, the cancer samples displayed a selective reduction in secondary bile acids that function as LIFR antagonists, together with a relative enrichment of primary bile acids, which, in contrast, lack these antagonistic properties. This imbalance between primary and secondary bile acids resulted in a significantly reduced ratio of LIFR antagonist to non-antagonist bile acids within the tumor microenvironment, a setting that may favor enhanced LIFR signaling. The functional classification of bile acids as endogenous LIFR antagonists is supported by experimental evidence demonstrating their ability to directly inhibit LIFR signaling in oncogenic contexts [23].

From a clinical perspective, the accumulation of primary bile acids was associated with worse overall survival, further supporting the biological relevance of bile acid-LIFR interactions in disease progression. Dysregulation of bile acid metabolism has also been implicated in systemic metabolic abnormalities and inflammatory responses associated with cancer cachexia [35]. Together, these data suggest that metabolic reprogramming of bile acid composition in gastric cancer may contribute to sustained activation of LIF/LIFR signaling by removing endogenous inhibitory constraints. Within this framework, the bile acid–LIFR–LIF axis should be regarded as a working model rather than a fully established mechanistic pathway.

The link between dysregulated LIF signaling and cachexia-related features observed in this study is particularly noteworthy. LIF is a pleiotropic cytokine signaling through a receptor complex involving LIFR and gp130, regulating inflammatory responses, cellular differentiation, and metabolic adaptation [36]. Both LIF and GDF15 have been implicated in appetite suppression, systemic inflammation, and muscle wasting in experimental models, and elevated circulating levels of these cytokines have been reported in cachectic cancer patients [37]. In preclinical studies, pharmacological blockade of the GDF15-GFRAL axis has been shown to reverse anorexia and muscle loss, providing direct evidence for its causal role in cancer-associated cachexia [6]. Within this framework, GDF15 may operate as components of a coordinated cytokine network activated in gastric cancer, contributing to anorexigenic and catabolic programs, without implying a defined upstream–downstream hierarchy. Importantly, while GDF15 shows a consistent association with adverse prognosis, LIF expression appears more closely related to body composition and inflammatory features. Notably, although not reaching statistical significance, higher LIF expression showed a trend toward poorer overall survival, as illustrated in Supplementary Figure S1, suggesting distinct but potentially converging roles of LIF and GDF15 in the cachexia phenotype. The strong correlation between these two factors, together with their shared associations with inflammation and body composition changes, supports the existence of a coordinated cytokine network associated with the cachexia phenotype in gastric cancer.

Collectively, our findings identify the LIF-GDF15 axis as a coordinated signaling network associated with tumor progression, metabolic remodeling, and systemic features of cancer-associated cachexia.

Importantly, the clinical associations observed in gastric cancer patients were supported by experimental evidence obtained in three-dimensional tumor models and paracrine co-culture systems. Gastric cancer spheroids displayed basal expression of both LIF and LIFR, consistent with the presence of an active autocrine signaling loop [38,39,40]. Exogenous LIF stimulation enhanced spheroid proliferative activity, as indicated by increased Ki67 expression, supporting a role for LIF signaling in promoting tumor aggressiveness. These findings align with previous reports implicating LIF in tumor growth and malignant progression and provide functional validation of its relevance in gastric cancer.

Beyond tumor-intrinsic effects, LIF-driven signaling exerted marked paracrine consequences on peripheral tissues involved in cachexia. Conditioned media derived from LIF-stimulated gastric cancer spheroids impaired myogenic differentiation in C2C12 cells, as reflected by reduced myotube diameter.

Notably, pharmacological inhibition of LIFR signaling attenuated LIF-induced transcriptional programs, including GDF15 expression as well as proliferation- and EMT-associated markers, supporting the dependence of these effects on LIFR activation (Supplementary Figure S4). In addition, fully differentiated C2C12 myotubes maintained under standard differentiation conditions exhibited markedly higher αSMA expression compared with myotubes exposed to conditioned media from gastric cancer spheroids, even in the absence of exogenous LIF stimulation (Supplementary Figure S4). These findings indicate that tumor-derived paracrine signals, including basal LIF activity, are sufficient to suppress physiological myogenic maturation programs.

These observations are consistent with a catabolic muscle phenotype and support the notion that tumor-derived LIF-related signals are consistent with a contribution to skeletal muscle wasting, a defining feature of cancer cachexia. While these experiments do not identify a specific soluble mediator, they support a tumor-driven paracrine effect associated with LIF-enriched signaling conditions.

In parallel, exposure of HepaRG cells to conditioned media from LIF-stimulated spheroids resulted in downregulation of key genes involved in bile acid synthesis and lipid metabolism, including *CYP7A1*, *FXR*, *FAS*, *PPARα*, and *SREBP1c*. These changes suggest that LIF-enriched tumor-derived factors negatively impact hepatic metabolic pathways, potentially contributing to systemic metabolic dysregulation. Together, these in vitro findings provide mechanistic support for the clinical and metabolic alterations observed in gastric cancer patients and reinforce the concept that tumor-derived LIF signaling is associated with multi-organ dysfunction characteristic of cancer-associated cachexia.

These observations have important clinical implications: (i) LIF and GDF15 may serve as biomarkers for disease severity and cachexia risk stratification in gastric cancer patients; (ii) therapeutic strategies aimed at modulating LIF/LIFR signaling, either directly or through metabolic interventions restoring endogenous LIFR antagonists, may represent a novel avenue to mitigate both tumor progression and cachexia [41].

This study has some limitations. The bile acid and body composition analyses were conducted in a limited subset of patients, and the observational nature of the clinical correlations precludes definitive conclusions regarding causality. Moreover, while our data strongly support an association between bile acid dysregulation, LIF signaling, and cachexia-related features, mechanistic validation in experimental models will be required to fully delineate the underlying pathways.

Therefore, although our findings highlight a coordinated involvement of LIF and GDF15 in cachexia-related phenotypes, they do not establish a causal hierarchy between these factors, nor do they demonstrate a direct causal role in driving cachexia.

In conclusion, this study identifies a previously underappreciated link between bile acid metabolism, LIF/LIFR signaling, and cancer-associated cachexia in gastric cancer. By integrating clinical, metabolic, and transcriptomic data, we propose a model in which tumor-driven alterations in bile acid composition are associated with sustained LIF signaling, in parallel with tumor aggressiveness and systemic metabolic derangement. These findings open new perspectives for biomarker development and therapeutic targeting in gastric cancer and its cachectic complications.

## 5. Conclusions

In conclusion, this study identifies a coordinated association between tumor-derived LIF signaling and GDF15 expression in gastric cancer and links this axis to cachexia-related clinical, metabolic, and body composition alterations. Integrated analyses of patient cohorts and experimental models indicate that LIF/LIFR activation is associated with tumor aggressiveness and exerts paracrine effects on skeletal muscle and hepatic metabolic pathways. While the present findings do not establish a causal hierarchy between LIF and GDF15, they support the relevance of the LIF-GDF15 network as a clinically meaningful signaling axis in gastric cancer and provide a rationale for future mechanistic and longitudinal studies.

## Figures and Tables

**Table 1:** **Antibodies used for immunofluorescence.** Primary and secondary antibodies used for immunofluorescence experiments, including source, catalog number, host species, and working dilution.

Target	Antibody	Supplier	Catalog No.	Host	Dilution
**α-Smooth Muscle Actin (αSMA)**	Alpha Smooth Muscle Actin	Invitrogen, Carlsbad, CA, USA	14-9760-82	Mouse	1:200
**LIF**	LIF Polyclonal Antibody	Invitrogen, Carlsbad, CA, USA	PA5-79600	Rabbit	1:200
**LIFR**	Anti-LIFR antibody	Abcam, Cambridge, UK	ab235908	Rabbit	1:200
**Ki67**	Recombinant Anti-Ki67 antibody	Abcam, Cambridge, UK	ab16667	Rabbit	1:200
**Secondary**	Goat anti-mouse IgG Alexa Fluor 488	Invitrogen, Carlsbad, CA, USA	A32723	Goat	1:200
**Secondary**	Goat anti-rabbit IgG Alexa Fluor 568	Invitrogen, Carlsbad, CA, USA	A11011	Goat	1:1000
**Secondary**	Goat anti-rabbit IgG Alexa Fluor 488	Abcam, Cambridge, UK	ab150077	Goat	1:200

**Table 2:** **Primers used for quantitative real-time PCR.** List of primers used for quantitative real-time PCR analysis, including gene name, species, and primer sequences.

ID Gene	Species	Sequence Forward	Sequence Reverse
*hRPLP0*	Homo sapiens	TaqMan Probe	
*hGDF15*	Homo sapiens	GAGCTGGGAAGATTCGAACA	AGAGATACGCAGGTGCAGGT
*hc-MYC*	Homo sapiens	TCGGATTCTCTGCTCTCCTC	TTTTCCACAGAAACAACATCG
*hSNAIL1*	Homo sapiens	ACCCACACTGGCGAGAAG	TGACATCTGAGTGGGTCTGG
*hLIF*	Homo sapiens	CCCTGTCGCTCTCTAAGCAC	GGGATGGACAGATGGACAAC
*hLIFR*	Homo sapiens	GCTCGTAAAATTAGTGACCCACA	GCACATTCCAAGGGCATATC
*mHprt1*	Mus musculus	CAGTCCCAGCGTCGTGATTA	TTTTCCAAATCCTCGGCATA
*mLif*	Mus musculus	GAATCAACTGGCACAGCTCA	GTTAGGCGCACATAGCTTTT
*mLifr*	Mus musculus	CTGGTGATCACGAAGTCACA	GATCTCGGGAGTCTCTGGAA
*mGdf15*	Mus musculus	CGAGAGGACTCGAACTCAGAA	GGTTGACGCGGAGTAGCAG
*hCYP7a1*	Homo sapiens	GACACACCTCGTGGTCCTCT	TTTCATTGCTTCTGGGTTCC
*hFXR*	Homo sapiens	GCAGCCTGAAGAGTGGTACTCTC	GCAGCCTGAAGAGTGGTACTCTC
*hFAS*	Homo sapiens	CGAAGAGGCGTGCCCTGAGCT	GCCGTAGTTGCTCTGTCCCG
*hSREBP1C*	Homo sapiens	GCAAGGCCATCGACTACATT	GGTCAGTGTGTCCTCCACCT
*hPPARα*	Homo sapiens	ATGGCATCCAGAACAAGGAG	ATGGCATCCAGAACAAGGAG

All TaqMan Probes were obtained from Life Technologies.

**Table 3:** Demographic and Clinical Data of GC patients.

ID	Age	Sex	Localization	Type of Gastrectomy	Lymphadenectomy	Lauren	pT	pN	pM	Stage	CY	Neoadiuvant
1	71	F	lower	subtotal	D2	intestinal	1 b	0	0	I	0	1
2	75	M	middle	subtotal	D2	intestinal	3	1	0	I	0	0
3	80	M	middle	subtotal	D2	intestinal	3	3 a	0	IV	1	0
4	83	F	lower	subtotal	D2	intestinal	3	1	0	II	0	0
5	83	M	upper	total	D1	intestinal	4 a	2	0	IV	1	0
6	76	M	upper	total	D2	intestinal	4 a	3 a	0	III	0	0
7	77	M	lower	subtotal	D2	intestinal	4 a	1	0	III	2	0
8	80	F	lower	subtotal	D1	intestinal	1 b	0	0	I	0	0
9	84	M	lower	subtotal	D1	intestinal	3	2	0	III	0	0
10	80	F	middle	subtotal	D2	diffuse	2	0	0	I	0	0
11	66	F	lower	total	D1	diffuse	3	2	1	IV	0	0
12	62	M	middle	total	D1	intestinal	1	0	0	I	0	0
13	83	M	lower	subtotal	D1	intestinal	2	0	0	I	0	0
14	75	M	lower	subtotal	D2	intestinal	4 a	0	0	II	0	0
15	75	F	lower	subtotal	D2	intestinal	2	1	0	II	2	0
16	78	F	lower	subtotal	D2	intestinal	3	0	0	II	2	0
17	57	M	upper	total	D2	diffuse	4 a	2	1	IV	0	1
18	72	M	upper	total	D2	intestinal	4 b	3 b	0	III	2	0
19	76	M	upper	subtotal	D2	diffuse	4 a	3 b	0	III	0	0
20	76	M	lower	subtotal	D2	intestinal	4 a	3 a	0	III	0	0
21	67	M	lower	subtotal	D2	intestinal	4 a	0	0	IV	1	0
22	82	F	lower	subtotal	D2	intestinal	3	3 b	1	IV	1	0
23	74	F	middle	subtotal	D2	diffuse	3	2	0	III	0	0
24	77	F	middle	total	D2	intestinal	3	3 a	0	III	0	0
25	78	F	lower	subtotal	D2	intestinal	3	3 a	0	III	0	0
26	88	F	lower	subtotal	D1	diffuse	3	3 a	0	IV	1	0
27	86	F	upper	total	D1	diffuse	4 b	0	0	III	2	0
28	78	M	lower	subtotal	D2	intestinal	3	2	0	III	0	0
29	63	M	lower	subtotal	D2	intestinal	3	3 a	0	III	0	0
30	79	F	lower	subtotal	D2	diffuse	4 a	3 b	0	III	0	0
31	76	M	upper	total	D2	intestinal	4 a	3 a	1	III	0	0
32	49	M	upper	total	D1	diffuse	3	3 b	1	IV	0	0
33	71	M	middle	subtotal	D2	intestinal	3	2	0	III	0	0
34	65	M	upper	total	D2	intestinal	4 b	1	1	IV	1	1
35	75	F	upper	total	D2	diffuse	4 a	3 b	0	III	0	0
36	67	M	middle	subtotal	D2	diffuse	2	2	0	IV	1	0
37	71	M	middle	subtotal	D2	diffuse	4 a	3 b	0	IV	1	0
38	76	M	lower	subtotal	D2	intestinal	2	0	0	I	0	0
39	45	M	lower	subtotal	D2	diffuse	3	1	0	II	0	1
40	61	M	middle	total	D2	intestinal	1 a	0	0	I	0	0
41	56	M	upper	total	D2	intestinal	3	1	0	II	0	1
42	62	M	lower	subtotal	D2	intestinal	1 b	0	0	I	0	1
43	79	M	middle	subtotal	D2	intestinal	2	3 a	0	III	0	1
44	76	F	lower	subtotal	D2	intestinal	1 b	0	0	I	0	1
45	79	M	lower	subtotal	D2	intestinal	3	2	0	III	0	0
46	70	M	upper	total	D2	intestinal	1 a	0	0	I	0	0
47	74	F	middle	total	D2	intestinal	2	0	0	II	0	1
48	80	F	lower	subtotal	D2	intestinal	1 b	0	0	I	0	0
49	72	M	middle	total	D2	intestinal	3	0	0	II	0	1
50	66	F	lower	subtotal	D2	intestinal	1 b	0	0	I	0	0
51	82	F	lower	subtotal	D1	intestinal	3	1	0	II	0	0
52	84	F	middle	total	D2	intestinal	4 a	3 a	0	III	0	0
53	63	F	lower	subtotal	D2	intestinal	0	0	0	0	0	0
54	75	M	lower	subtotal	D2	intestinal	3	1	0	II	0	0
55	64	M	lower	subtotal	D2	intestinal	2	0	0	I	0	1

pT = pathological Tumor; pN = pathological Nodes; pM = pathological Metastasis; CY = Cytology. a/b = TNM subclassification. Tumor staging was defining according to the TNM classification as recommended by the ESMO Clinical Practice Guideline for gastric cancer [28].

## Data Availability

The data generated in this study are publicly available The transcriptomic data obtained from the experimental cohort are publicly available in Mendeley Data, Mendeley Data, V1, doi: 10.17632/j46b34szhw.1 data that support the findings of this study were ob-tained from The GSE66229 Dataset from ACRG at [https://www.ncbi.nlm.nih.gov/geo/query/acc.cgi?acc=GSE66229], accessed on 31 January 2023. The TCGA-STAD at [https://portal.gdc.cancer.gov/].

## Supplementary Materials

The following supporting information can be downloaded at: https://www.mdpi.com/article/10.3390/cells15040355/s1, Figure S1: Transcriptome analysis of non-neoplastic and neoplastic tissues was carried out in 61 GC patients, who underwent surgical resection at Perugia University Hospital; Figure S2: Negative controls for immunofluorescence staining of MKN45 gastric cancer spheroids. Representative images of three-dimensional MKN45 spheroids processed for immunofluorescence in the absence of primary antibodies. Nuclei were counterstained with DAPI (blue). Red and green channels correspond to secondary antibodies only and show no specific signal, confirming the absence of nonspecific staining. Merged images are shown in the bottom row. Scale bars as indicated; Figure S3: (A) Representative Western blot analysis showing basal protein expression of LIF and LIF receptor (LIFR) in MKN45 cells. Vimentin was used as control. (B) Representative intracellular flow cytometry (IC-FACS) analysis of Ki67 expression in MKN45 cells left untreated (NT) or stimulated with recombinant human LIF (10 ng/mL). Overlaid histograms show Ki67 fluorescence intensity. Quantification of the percentage of Ki67-positive cells is reported on the right. Data are expressed as mean ± SEM; Figure S4: (A) 2D Chemical structure of LRI-305, a small-molecule antagonist of the leukemia inhibitory factor receptor (LIFR). (B) In vitro potency of LRI-305 as assessed by AlphaScreen binding assay, STAT3 transactivation assay, and MTS cell viability assay. Data are reported as IC_50_ values (µM, mean ± SEM). (C) MKN45 was exposed to 10 ng/mL of LIF alone or in combination with 10 μM of LRI305. Cells left untreated (NT) were used as control. Relative mRNA expression of GDF15, Ki67a and SNAIL1. Data are expressed as mean ± SEM (*n* = 3). Each value is normalized to *RPLP0* and is expressed relative to those of NT, which are arbitrarily set to 1.
