# Supplementary material for: Tumor-Derived LIF Promotes GDF15-Driven Cachexia and Adverse Outcomes in Gastric Cancer
Source: Cells. 2026 Feb 16;15(4):355. doi: 10.3390/cells15040355 (PMC12938927; doi:10.3390/cells15040355)

Supplementary Figure Legends

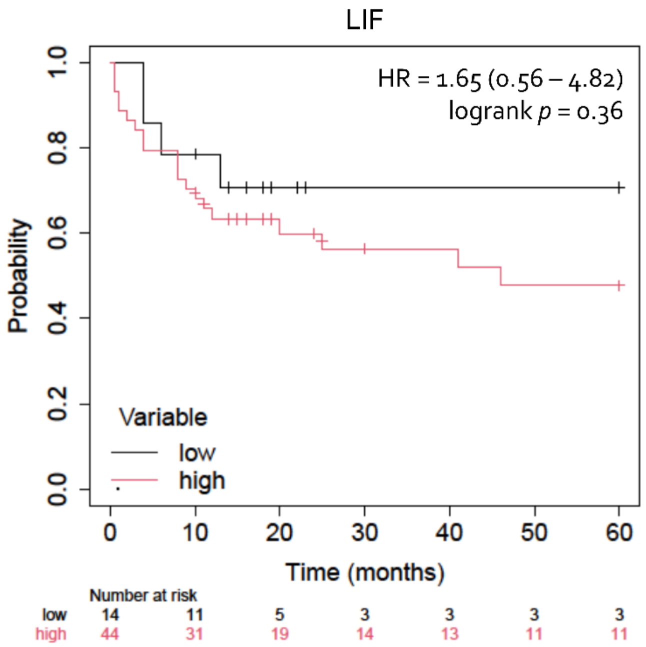

Supplementary Figure s1. Transcriptome analysis of non-neoplastic and neoplastic tissues was carried out in 61 GC patients, who underwent surgical resection at Perugia University Hospital. Kaplan Meyer analysis of LIF. *p < 0.05.

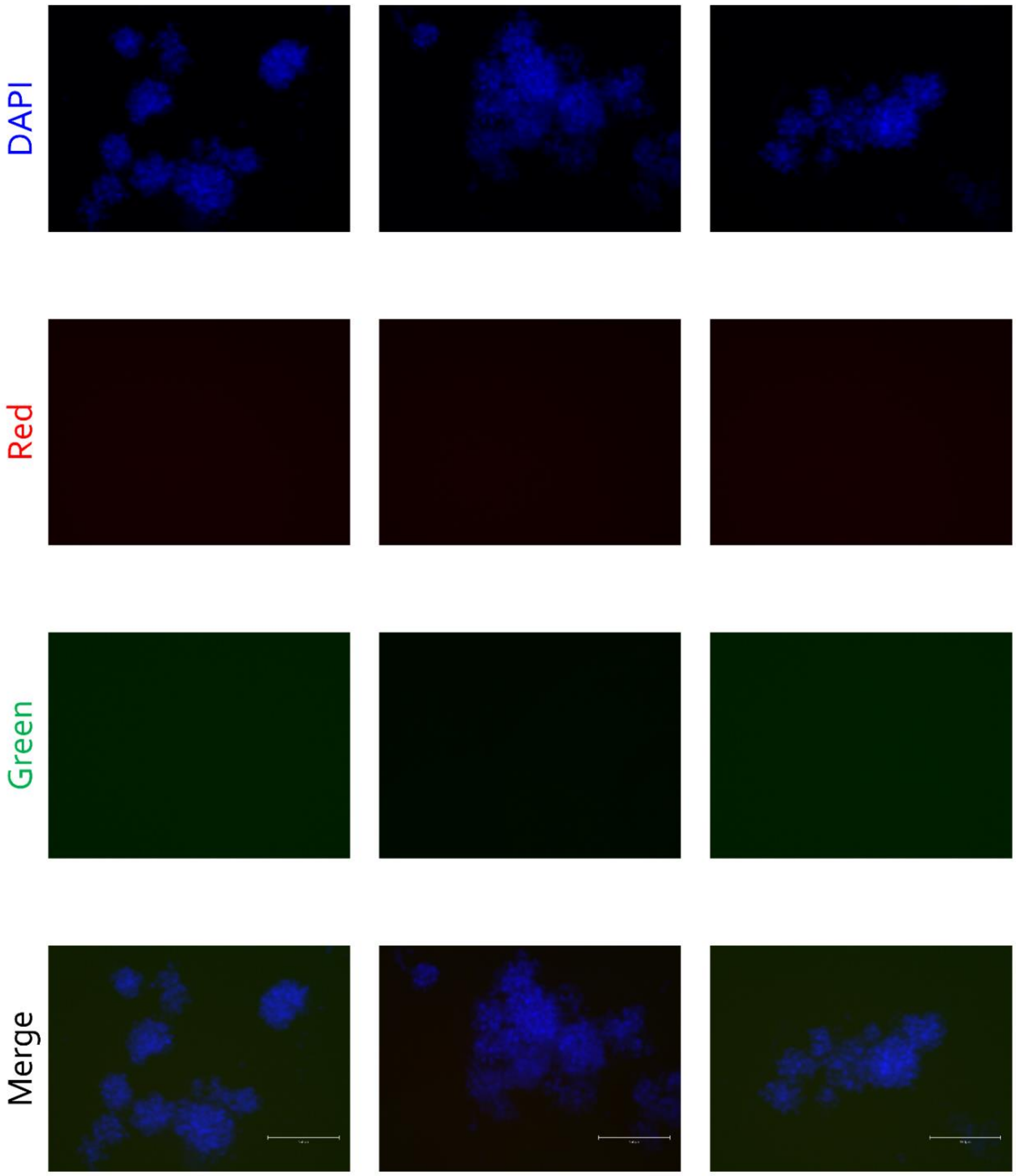

Supplementary Figure S2. Negative controls for immunofluorescence staining of MKN45 gastric cancer spheroids. Representative images of three-dimensional MKN45 spheroids processed for immunofluorescence in the absence of primary antibodies. Nuclei were counterstained with DAPI (blue). Red and green channels correspond to secondary antibodies only and show no specific signal, confirming the absence of nonspecific staining. Merged images are shown in the bottom row. Scale bars as indicated.

A

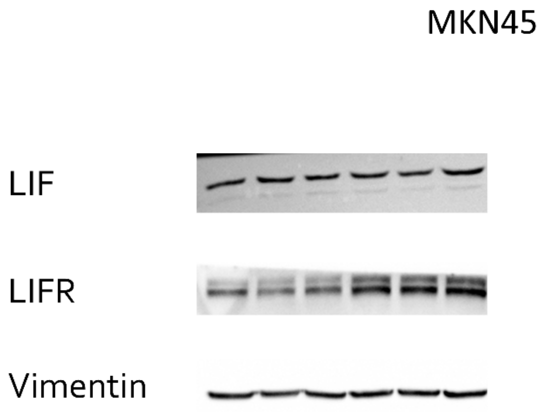

B

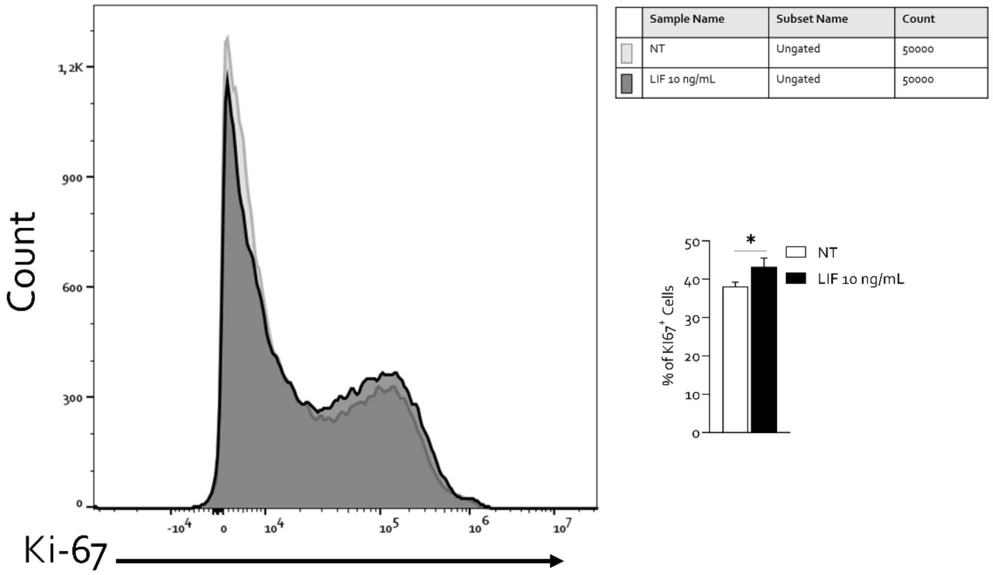

Supplementary Figure S3. A) Representative Western blot analysis showing basal protein expression of LIF and LIF receptor (LIFR) in MKN45 cells. Vimentin was used as control. **B)** Representative intracellular flow cytometry (IC-FACS) analysis of Ki67 expression in MKN45 cells left untreated (NT) or stimulated with recombinant human LIF (10 ng/mL). Overlaid histograms show Ki67 fluorescence intensity. Quantification of the percentage of Ki67-positive cells is reported on the right. Data are expressed as mean ± SEM. * $p < 0.05$.

A

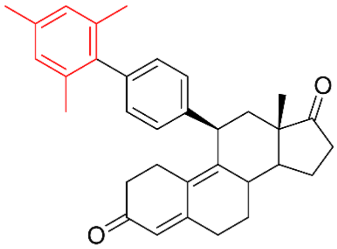

LRI 305

B

Assay	IC_{50} (μM)
AlphaScreen	0.86 ± 0.239 [31]
STAT3 Transactivation	2.9 ± 0.9
MTS	0.7 ± 0.19

C

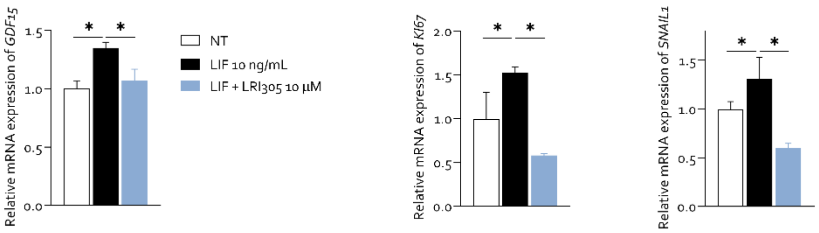

Supplementary Figure S4. A) 2D Chemical structure of LRI-305, a small-molecule antagonist of the leukemia inhibitory factor receptor (LIFR). **B)** In vitro potency of LRI-305 as assessed by AlphaScreen binding assay, STAT3 transactivation assay, and MTS cell viability assay. Data are reported as IC_{50} values (μM, mean ± SEM). **C)** MKN45 was exposed to 10 ng/mL of LIF alone or in combination with 10 μM of LRI305. Cells left untreated (NT) were used as control. Relative mRNA expression of GDF15, Ki67a and SNAIL1. Data are expressed as mean ± SEM (n=3). Each value is normalized to *RPLPo* and is expressed relative to those of NT, which are arbitrarily set to 1. *p < 0.05.

Original Western Blot

Vimentin

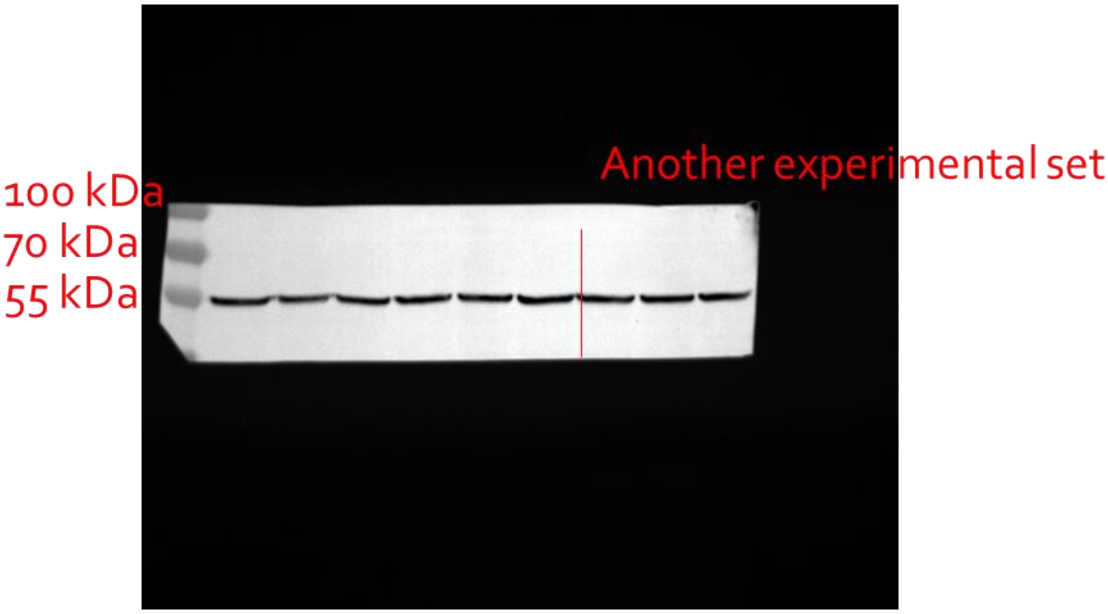

LIF

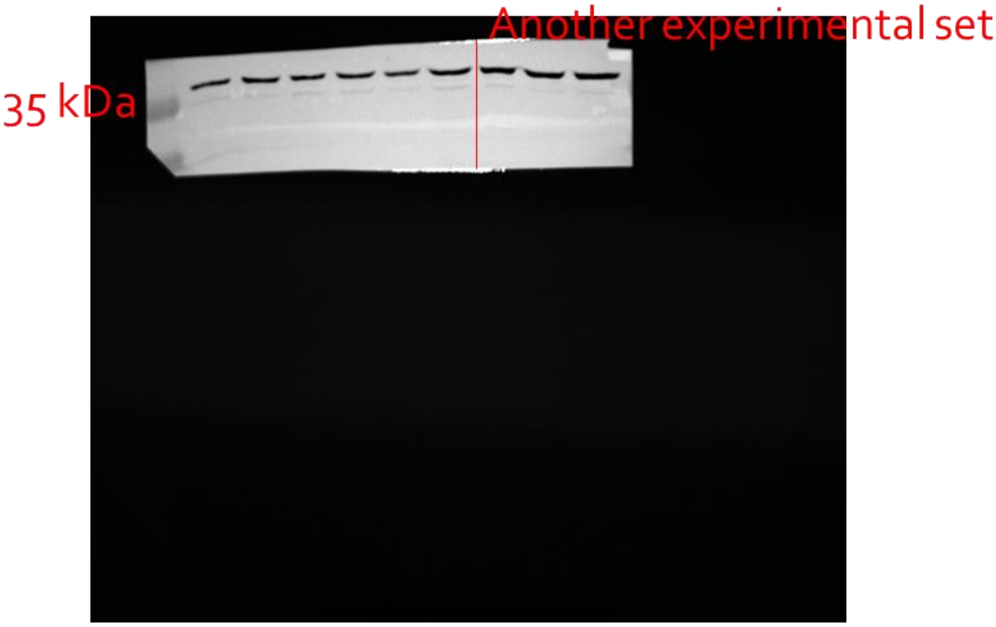

LIFR

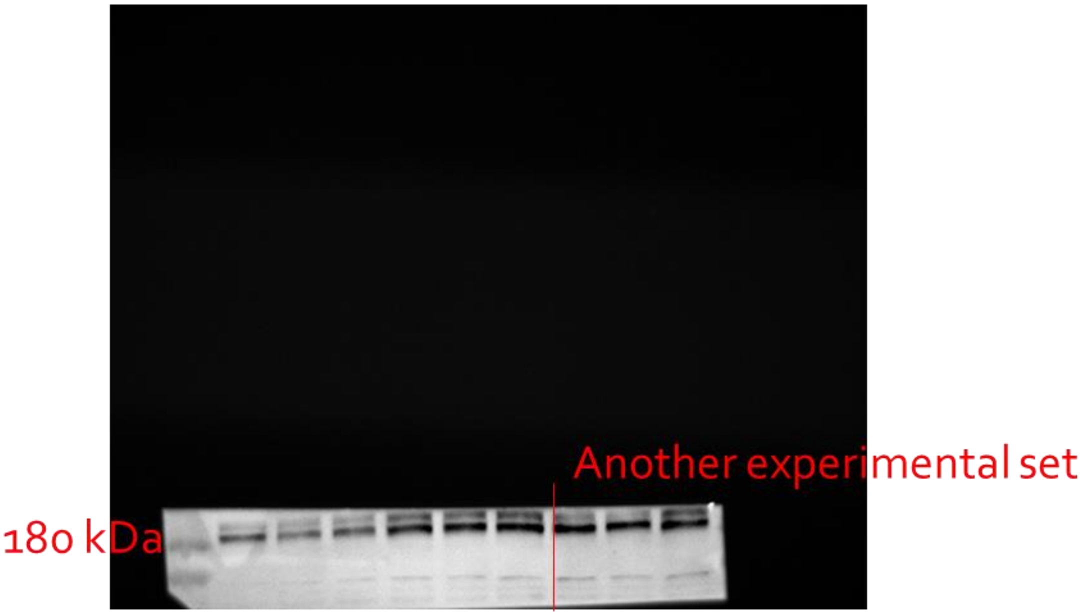

Supplement: Supplementary file 1 [file cells-15-00355-s001.zip › cells-4098648-supplementary.pdf]
